# Whole-Genome Sequence of Lactobacillus plantarum SPC-SNU 72-2 as a Probiotic Starter for Sourdough Fermentation

**DOI:** 10.1128/MRA.00708-20

**Published:** 2020-10-15

**Authors:** Seul-Ah Kim, Geonhee Kim, Bo Bo, Sangmin Shim, Deukbuhm Lee, Shin Dal Kang, Jin-Ho Seo, Nam Soo Han

**Affiliations:** a Brain Korea 21 Center for Bio-Resource Industry, Division of Animal, Horticultural, and Food Sciences, Chungbuk National University, Cheongju, Republic of Korea; b Research Institute of Food and Biotechnology, SPC Group, Seoul, Republic of Korea; University of Maryland School of Medicine

## Abstract

We report the whole-genome sequence of Lactobacillus plantarum SPC-SNU 72-2, a probiotic starter for sourdough. Genome sequencing was completed using the Pacific Biosciences RS II and Illumina platforms. This study will facilitate the understanding of microbial characteristics of *L. plantarum* SPC-SNU 72-2 and its roles during sourdough fermentation.

## ANNOUNCEMENT

Lactobacillus plantarum is a Gram-positive, facultative, anaerobic bacterium which has been used as a food supplement or starter because of its proposed health-promoting properties (1, 2). *L. plantarum* in sourdough fermentation is important for producing acids and exopolysaccharide contributing to physical properties (3, 4). In a previous study, *L. plantarum* SPC-SNU 72-2 was isolated by spreading serially diluted kimchi on MRS medium at 37°C for 2 days and, finally, was deposited in the Korean Collection for Type Cultures (KCTC, accession no. KCTC 13315BP). The strain has tolerance to acid and bile salt, the ability to adhere to epithelial cells, and putative immunostimulatory activity (5). Here, we report the whole-genome sequence of *L. plantarum* SPC-SNU 72-2, a probiotic starter for sourdough.

The genomic DNA of *L. plantarum* was extracted from overnight culture in MRS medium (Difco, Detroit, MI, USA) at 37°C using a DNA minikit (BioFact, Daejeon, Republic of Korea). Genome sequencing was completed using the Pacific Biosciences (PacBio, USA) RS II and Illumina (USA) HiSeq 2500 instruments at Macrogen (Seoul, Republic of Korea). The sequencing libraries were generated with a SMRTbell template prep kit v3.0 (PacBio) for PacBio RS II and a TruSeq Nano DNA kit (Illumina) for Illumina following the manufacturer’s protocols. The quality of the raw data sequences was checked using FastQC v0.11.9 (6). First, the reads (112,011), with an average length of 11,244 bp (1,259,535,953 total subread bases), were generated with the PacBio system, and *de novo* assembly was conducted using Canu v1.7 software (7). When both ends of the contig overlapped, the contig was regarded as a circular form, and overlapped regions were manually trimmed. Among the contigs, if the query cover was higher than 80% in the BLASTN v2.7.1+ results compared with those of other *L. plantarum* strains in the NCBI database and its size was highly similar, it was classified as a chromosome or plasmid. The assembly resulted in a single circular contig, which was rotated to allow all genomes to start at the same site. Second, an Illumina HiSeq 2500 system was applied to construct contigs more accurately. For this, HiSeq reads were subjected to a final polishing step to ensure genome sequence accuracy using Pilon v1.21 (8). In the case of the Illumina platform, 4,473,248 total reads (675,460,448 total read bases) were generated, 4,383,091 of which (97.98%) were mapped (mapped site, 99.99%) with a coverage depth of 201.29-fold. The assembly quality was checked by comparison between predicted genes from the genome assembly and Benchmarking Universal Single-Copy Orthologs (BUSCO) v3.0 (quality score, 99.19%) (9). Finally, the genome sequence of *L. plantarum* SPC-SNU 72-2 was annotated using the NCBI Prokaryotic Genome Annotation Pipeline (PGAP) v4.11 (10). All software was run with default settings unless otherwise specified.

The final genome assembly contained one full circular chromosome and four plasmids, and the genome contained 3,253,666 bp with a G+C content of 44.46%. In detail, the chromosome has 3,037,092 bp with a 44.82% G+C content, and the four plasmids have 75,282 bp with a 39.18% G+C content, 51,115 bp with a 40.29% G+C content, 45,820 bp with a 39.76% G+C content, and 44,357 bp with a 38.79% G+C content, respectively. This strain has a total of 3,104 genes, which are composed of 2,907 protein coding sequences (CDSs), 93 RNA genes, and 104 pseudogenes. This study will facilitate the understanding of microbial characteristics of *L. plantarum* SPC-SNU 72-2 and its roles during sourdough fermentation.

### Data availability.

The genome sequences for *L. plantarum* SPC-SNU 72-2 have been deposited in GenBank under accession no. CP050805.1 for the chromosome and CP050806.1, CP050807.1, CP050808.1, and CP050809.1 for the plasmids. The raw sequence reads have been deposited in the SRA under accession no. SRR11776882 (HiSeq reads) and SRR11929935 (PacBio reads).
